# Uncovering the hidden bacterial ghost communities of yeast and experimental evidences demonstrates yeast as thriving hub for bacteria

**DOI:** 10.1038/s41598-021-88658-x

**Published:** 2021-04-30

**Authors:** B. Indu, Tallapragada Keertana, Sahu Ipsita, Uppada Jagadeeshwari, Chintalapati Sasikala, Chintalapati Venkata Ramana

**Affiliations:** 1 Department of Plant Sciences, School of Life Sciences, University of Hyderabad, Hyderabad, India; 2 Bacterial Discovery Laboratory, Centre for Environment, Institute of Science and Technology, J.N.T. University Hyderabad, Hyderabad, India

**Keywords:** Microbiology, Plant sciences

## Abstract

Our major concern was to address “yeast endobacteria” which was based on a few reports in the recent past where bacteria may find yeast as a niche for survival. In this study, we report the microbiota of twenty-nine axenic yeast cultures recovered from different habitats based on their 16S rRNA gene-amplicon metagenomes. Yeasts were identified based on D1/D2 or ITS gene sequences. Bacterial diversity was widespread, varied and rich among all yeasts except for four strains. Taxa belonging to the phylum *Firmicutes*, *Proteobacteria*, *Actinobacteria* and *Bacteroidetes* and the genera; *Streptococcus*, *Propionibacterium* were common to all the yeasts. *Candida tropicalis* was used as a model organism to confirm bacteria through fluorescence in situ hybridization (FISH), isolating and re-introducing the isolated bacteria into the yeast. FISH analysis confirmed the endobacteria of *C. tropicalis* and we have successfully isolated four bacteria only after lysis and disruption of yeast cells. These bacteria were identified as species of *Pseudomonas*, *Chryseobacterium*, *Lysinibacillus* and *Propionibacterium*. Guestimates indicate 95% of bacterial species of *C. tropicalis* are yet-to-be-cultivated. We have successfully reintroduced mCherry tagged *Pseudomonas* into *C. tropicalis*. Also, auto-fluorescent *Prochlorococcus* and *Rhodopseudomonas* could be introduced into *C*. *tropicalis* while mCherry tagged *E. coli* or *Salmonella* could not be introduced. FISH analysis confirmed the presence of both native and infected bacterial cells present in *C. tropicalis*. Our findings unveil the insights into the ghost microbiota associated with yeast, which otherwise are considered to be axenic cultures. Their inherent occurrence, together with co-cultivation experiments under laboratory conditions suggests that yeasts are a thriving hub for bacterial communities.

## Introduction

There is a saying among microbial ecologists that no organism lives in isolation. The importance of interactions and relationships between different organisms, even if it is a unicellular one cannot be ignored^1^. Of these, bacteria-fungus interactions (BFI) have been only recently investigated in detail, giving us a different perspective into the nature and impacts of these relationships^2,3^. They are very important because fungi and bacteria are commonly found together in many natural communities^2–7^. Studies on BFI have shown the role of both organisms in influencing each other’s biology as well as their influence on the ecosystem^1–5^. For instance, the bacterial endosymbionts of soil fungus *Rhizopus* control the molecular reins of the fungus to become heritably transmitted^6^. Other interesting cases include the recently uncovered relationships between plant-associated filamentous fungi and their endobacteria, some of which were shown to be uncultivable^2,3,7^. Such interesting cases show the newly uncovered facet of microbial interactions, where dynamics previously thought to exist between certain partners (for example plant-fungus) have now been extended to the bacterial endosymbionts (for example, plant-fungus-endobacteria). However, bacterial interactions (particularly endobacteria) with yeast, remains obscure and not much is still known.

Yeasts engage in a wide variety of relationships^8^ such as predation^9^ and processes like autophagy^10^. Documented shreds of evidence show that many times, yeasts and bacteria co-exist in the same environment^11^ and yet, their interactions have not been studied in depth. Fungal microbiomes are becoming a thriving area of research, showing how distinct each microbiome is to the fungal host^2,3,7^. It has been speculated that such fungal microbiomes may contribute to the host biology just like the animal and plant microbiomes do^2^. Their roles are different in different fungi. For example, *Burkholderia rhizoxinica* has a positive effect on *Rhizopus* whereas *Candidatus* Moeniiplasma glomeromycotorum (CaMg) has a negative impact on Mucoromycota^3^. Well-studied cases of fungal endobacteria to date have been mostly restricted to few fungal hosts like the AMF and other plant-associated filamentous fungi^2–4,6^. Although some research related to bacterial ‘endosymbionts’ of yeasts is still underway^12–17^, such cases are sparse in number and can be counted on the fingers. One of the earliest reports among these was of a possible symbiotic relationship between *Candida tropicalis* and *Microbacterium* sp.^17^. Other reports demonstrated *Helicobacter* cells inside yeast vacuoles by immunofluorescence, where vacuoles were considered as a specialized niche for such bacteria^12,14^. Localization of *Staphylococcus* in yeast vacuole was also similarly shown^15^. Work on *Saccharomyces* and the endosymbiont *Wolbachia*, demonstrated the latter’s infection in the yeast, allowing for the study of host-endosymbiont dynamics^16^. Yeast-bacterial interactions, particularly the endobacteria phenomenon was speculated as potentially important to nectar research, where both these microorganisms play a key role in the ecology and biochemistry of flower nectar^5^. Similarly, the yeast *Rhodotorula mucilaginosa* was shown to harbor endosymbiotic *Pseudomonas stutzeri*^18^. This yeast-endobacteria system has been implicated in interactions with rice plant that hosts the yeast, wherein these microorganisms collectively aid in nitrogen fixation^19^. Additionally, there has been growing interest to artificially generate yeast-endobacteria systems to understand the endosymbiosis theory^20,21^. Such simulations aim to fuel further research on endosymbiosis and to allow the elucidation of events involved. We explored the fascinating occurrence of bacteria associated with yeasts, which started with our serendipitous observation of moving Bacteria-Like-bodies (BLBs) inside yeast cells. We begin by presenting the diversity of yeasts and their bacteria. We also made attempts to isolate some of these bacteria and infect them to the yeast, in order to similarly relate to Koch’s postulates.

## Methods

### Isolation, identification and preservation of yeast

Environmental samples collected from different geographical regions of India were used as sources for yeast isolation which include soils, fruits and other biological sources (Table 1). Samples were inoculated in Yeast Peptone Dextrose (YPD) broth (HIMEDIA, M1363) containing chloramphenicol (100 µg mL^−1^) and kept for enrichment for 48–72 h at 30 °C. Enrichment cultures were streaked onto YPD agar with chloramphenicol and incubated at 30 °C for 4–5 days. Isolated colonies were repeatedly streaked to obtain axenic cultures which were preserved as glycerol stocks (50% v/v) at − 20 °C. Purity of the yeast cultures was confirmed by streaking the yeast on nutrient agar and simultaneously observing under microscope. A total of 29 yeast strains were isolated, purified and preserved. They were named in the JY series starting from JY101.

**Table 1:** Source, highest identity and accession numbers of D1/D2, ITS or metagenome of yeasts used in this study.

Source	Strain	Similarity with the nearest yeast	D1/D2 or ITS accession	Metagenome accession number of the yeast
Soil	JY101	*Candida tropicalis* CBS 94^T^	LT999794	SRR7746671
Date fruit	JY106	*Candida tropicalis CBS 94^T^	LT719072	SRR7752355
Lichen	JY107	*Candida tropicalis CBS 94^T^	LT719073	SRR7752303
Leaf litter	JY108	*Candida tropicalis CBS 94^T^	LT719074	SRR7752354
Citrus fruit	JY113	*Candida tropicalis CBS 94^T^	LT795049	SRR7757783
Decomposed wood	JY114	*Candida tropicalis CBS 94^T^	LT795050	SRR7757789
Biofilm	JY125	*Candida tropicalis* ATCC 750^T^	LT838873	SRR7762712
Carve soil	JY134	*Candida tropicalis* CBS 94^T^	LT995997	SRR7774106
Sewage water	JY103	*Candida metapsilosis CBS 10907^T^	LT719069	SRR7752356
Muskmelon	JY110	*Candida intermedia CBS 572^T^	LT795046	SRR7757784
Brown algae *Gracillaria* sp.	JY121	*Candida suratensis* CBS 10928^T^	LS992563	SRR7760802
Brown algae *Gracillaria* sp.	JY124	*Candida suratensis* CBS 10928^T^	LT840079	SRR7762711
Kadam fruit	JY135	*Candida ampae* CBS 7872	LT996821	–
Gooseberry	JY105	*Pichia kudriavzevii CBS 5147^T^	LT719071	SRR7752353
Idly batter	JY112	*Pichia kudriavzevii CBS 5147^T^	LT795048	SRR7757792
Sugarcane	JY116	*Pichia kudriavzevii CBS 5147^T^	LT795052	SRR7757791
Lichen	JY131	*Pichia kudriavzevii* NRRL Y-5396^T^	LT962958	SRR7774038
Mushroom	JY129	*Pichia kudriavzevii* NRRL Y-5396^T^	LT962960	SRR7762710
Fruit *Neolamarckia cadamba*	JY136	*Pichia kluyveri* NRRL y-11519^T^	LT996820	SRR7774105
Idly batter	JY104	*Meyerozyma guilliermodii CBS 2030^T^	LT719070	SRR7752352
Citrus fruit	JY117	*Meyerozyma carribicca CBS 9966^T^	LT795053	SRR7757782
Grapes	JY102	*Hanseniaspora guilliermondii* CBS 465^T^	LT999810	SRR7752479
Soil	JY109	*Rhodotorula mucilaginosa CBS 316^T^	LT795045	SRR7752357
Algal growth	JY127	*Rhodotorula mucilaginosa* CBS 316	LT799394	–
Mushroom	JY132	*Rhodotorula mucilaginosa* CBS 316	LT962959	–
Lake sediment	JY143	*Rhodotorula mucilaginosa* CBS 316	LT996827	–
Pine tree bark	JY119	*Zalaria obscura* DAOMC 250849^T^	LT840078	SRR7757780
Mushroom	JY130	*Debaryomyces prosopidis* JCM 9913^T^	LT962955	SRR7762713
Soil	JY133	*Sporidiobolus pararoseus* CBS 491^T^	LT962957	

–, not yielded results.

*Indicates the identification based on ITS sequencing.

### Isolation of genomic DNA, PCR amplification and identification of yeast

Genomic DNA of all the yeast strains was isolated using the method described by Hoffman^22^. Briefly, cells were lysed with Triton-X 100 and glass beads. DNA was extracted using phenol–chloroform-isoamyl alcohol and eluted with Tris EDTA buffer. For the identification of yeast, internal transcribed spacer (ITS) region was amplified with ITS1 (5′-GTCGTAACAAGGTTTCCGTAGGTG-3′) and ITS4 (5′-TCCTCCGCTTATTGATATGC-3′) primers and D1/D2 domain of 26S rRNA gene was amplified using NL1 (5′-GCATATCAATAAGCGGAGGAAAAG-3′) and NL4 (5′-GGTCCGTGTTTCAAGACGG-3′) primers respectively. DNA sequencing of the amplified regions were outsourced to AgriGenome Labs Pvt Ltd, India. BLAST analysis in the NCBI database was carried out for the sequences and identification of yeast was performed using the YeastIP database and CBS database^23^ (http://www.westerdijkinstitute.nl). All the sequences were submitted in the NCBI database and the accession numbers are given in Table 1.

### Staining and microscopy

Yeast cells were observed under phase contrast (Leica DFC295) microscopy where moving bacteria like bodies (BLBs) in the yeast cell were seen. To stain and observe these BLBs, ViaGram (Invitrogen, V7023) kit containing DAPI, SYTOX and Texas Red conjugated with wheat germ agglutinin (WGA) was used. Yeast cells were washed thrice with BSA-0.9% NaCl buffer and then incubated first with Texas Red for 15 min. Excess stain was removed by washing the incubated cells with BSA-0.9% NaCl buffer. To the washed cells, 2 μL mixture of DAPI and SYTOX (prepared according to the kit instructions) was added and incubated for 15 min in the dark. Cells were then observed under confocal microscope (Zeiss LSM880) which showed fluorescent red-stained BLBs under the excitation/emission maxima set to ~ 595/615 nm.

### Purity of yeast cultures, 16S rRNA gene metagenome analysis of V1–V3 region

The following protocols were strictly followed to ensure absence of extracellular bacteria before DNA extraction. Yeast strains were cultured on various bacterial media [Luria broth (HIMEDIA, M575), *Thiobacillus* agar (HIMEDIA, M788), Mueller Hinton agar (HIMEDIA, M173)] to confirm bacterial contamination. Contamination was also checked by observing yeast cells under phase contrast microscope and confocal microscope after staining the cells with Texas Red. In addition, the purity of yeast cultures was further confirmed by repeated subculturing in YPD media containing chloramphenicol (100 µg mL^−1^). DNA was extracted from the yeast cells by the method described by Hoffman^22^, from equivalent biomass of all yeast strains grown in YPD broth harvested at OD of 0.8. Amplification and sequencing of the V1–V3 region of the 16S rRNA gene were outsourced to RTL Genomics, USA and Eurofins Scientific, India, which was performed on Illumina MiSeq platform. As a control, samples without yeast biomass were also processed for DNA extraction protocol and sequencing. The analysis and taxonomy assignment to the sequences were done using mothur software package, version 1.41.1^24^. The protocol described by Schloss et al*.* for analysis of MiSeq data was followed^25^. First, the paired-end reads were assembled into contigs and screened for quality and removal of chimeras. The minimum length of sequences was restricted to 350 bp and the maximum length was limited to 550 bp to correspond to the size of the V1–V3 region of the 16S rRNA gene. The screened sequences were aligned to the SILVA 16S rRNA gene sequence database^26^. Sequences were again evaluated for chimeras, ambiguous, homopolymers and undesirable sequences such as those belonging to organelles, Archaea, eukaryotes were removed.

Aligned sequences were clustered into operational taxonomic units (OTUs) using 0.03 divergence cut-off. The raw sequences were submitted in the NCBI database and the accession numbers are given in Table 1 against each yeast strain. Diversity and comparison of the data along with graphical representation was done using Microsoft Excel and web-based tools. The MicrobiomeAnalyst online tool^27^ was used to filter data for the low count and low variance and normalize it by total sum-scaling for alpha and beta diversity measurements. Data was also rarefied as recommended to minimum library size. The Chao-1 index was used to measure alpha diversity and species richness with the statistical method set to T-test/ANOVA for significance testing. Bray–Curtis index calculated for beta diversity assessment among the samples was visualized as Principal Coordinates Analysis (PCoA) plots. Permutational multivariate analysis of variance was used to test the significance of the index. The common and unique features (phyla and genera) were evaluated visually in the form of Venn diagrams constructed using the InteractiVenn web-based tool^28^. Heatmaps were created using the Heatmapper online tool^29^ with the normalized data. Row and column clustering using average linkage was applied by Euclidean measurement of the distances. PAST V3.26 software was used to construct the rarefaction curves^30^.

### Isolation and cultivation of bacteria associated with yeast

As the metagenome results revealed the presence of more than one type of bacteria with the yeast cell, we used several approaches to isolate these bacteria from axenic yeast culture. These included zymolase treatment which degrades the cell wall of yeast and shearing of yeast cells via vortexing with glass beads. In the latter method, *Candida tropicalis* was grown in nutrient broth containing 100 µg mL^−1^ of chloramphenicol for about 98 h. Cells were centrifuged and sterile glass beads were added. This was vortexed vigorously to shear the yeast cells. The sheared yeast cells were centrifuged at 6000 × g for 5 min for settling down the yeast cell debris. Being lighter in weight, bacterial cells were presumed to remain in the supernatant. Hence, the supernatant was serially diluted up to 10^−5^ dilution and plated onto a nutrient agar plate containing cycloheximide (200 µg mL^−1^). Plates were then incubated at 30 °C for 1 week. Cultures with yellow and white color colonies were seen on the plates which were further purified by repeated sub-culturing. DNA of the purified colonies was extracted and the 16S rRNA gene for each isolate was amplified using F27 and R1525 primers as described earlier^31^. The amplified product was sent for sequencing to Agrigenome Labs Pvt Ltd, India. The sequenced product was searched with BLAST in EzBiocloud to get the identity of the nearest taxa.

### Fluorescence in situ hybridization

Fluorescence in situ hybridisation (FISH) is a powerful tool to enumerate and specify the bacteria in environmental samples or mixed cultures^32^. This protocol uses the oligonucleotide probe targeting the 16S rRNA of bacteria. Briefly, 2 mL of overnight grown yeast cells were centrifuged at 13,000 rpm for 5 min. Supernatant was discarded and the cells were fixed in 1 mL of fixative solution (4% formaldehyde in PBS buffer). Fixed cells were incubated for 3 h at room temperature and then centrifuged at 13,000 rpm for 5 min. The pellet obtained was resuspended in 50% ethanol and incubated for 5 min at room temperature. Resuspended pellet was centrifuged at 13,000 rpm for 5 min. Pellet was again washed with 80%, 95% ethanol and air dried the cell by placing it in speed vacuum for 10 min. 500 μL of hybridisation buffer (Solution of 20 mM TrisCl, 0.9 M NaCl, 0.01% SDS in 40% formamide) was added to dried cells and incubated at 37 °C for 30 min thereby firming pre-hybridisation mixture. 10 μL of bacterial specific probe EUB338-Cy3 (10 pmol μL^−1^ of working stock with sulfoindocyanine dye Cy3) was added to 50 μL of the pre-hybridisation mixture and incubated for 24 h at 50 °C. After 24 h, cells were centrifuged at 13,000 rpm for 5 min and washed thrice in 0.1X SSC buffer. The final pellet was dissolved in 20 μL of 0.1X SSC buffer. Slides were prepared and observed under confocal microscope with emission wavelength at 499–542 nm and excitation at 543 nm.

### Fluorescence labelling of *Pseudomonas stutzeri *JC703, *Escherichia coli* DH5α and *Salmonella typhimurium*

*Pseudomonas stutzeri* JC703, *Escherichia coli* DH5α and *Salmonella typhimurium* were tagged with fluorescent protein mCherry contained in a plasmid carrying antibiotic cassette for its maintenance in bacteria. Here, pBBRMCS-5 was used as the cloning vector which has a broad host range for Gram-negative bacteria carrying a gentamycin resistant cassette. The mCherry gene was cloned into pBBRMCS-5 plasmid under the constitutive expression of *tac* promoter as described earlier^33^. The constructed plasmid pBBRMCS-5 harboring the mCherry fluorescent tag and gentamycin resistant gene cassette was then transformed in the *P*. *stutzeri* JC703, *E. coli* DH5α and *S. typhimurium*.

Transformation of *P. stutzeri* JC703 was done using the heat shock protocol described by Feng and co-workers^34^, with a few modifications. Briefly, 100 μL of overnight grown *P. stutzeri* JC703 was inoculated in 10 mL of Luria broth (HIMEDIA) and incubated at 30 °C for 2–3 h until the OD reached 0.4–0.5. The culture was then kept on ice for 30 min followed by centrifuging 2 mL of culture at 7000 × g for 10 min. The pellet was washed three times in water and dissolved in 1 mL of 0.1 M CaCl_2_·2H_2_O (Sigma). Cells were incubated on ice for 1 h and centrifuged at 7000 × g for 10 min. The pellet was dissolved in 1 mL of a solution containing 0.7 mL of CaCl_2_·2H_2_O and 0.3 mL of 50% glycerol. The above steps were performed to make the cells competent to receive foreign DNA. The competent *Pseudomonas* cells were then used for transformation with the pBBRMCS plasmid. Competent *P. stutzeri* JC703 cells were incubated with the plasmid on ice for 30 min followed by heat shock at 42 °C for 6 min. Cells were immediately transferred to the ice for 3 min and then incubated in 0.9 mL of Luria broth (HIMEDIA) at 30 °C for 2 h. Cells were centrifuged and 0.9 ml of the supernatant was discarded and the remaining was spread onto LB agar plates containing gentamycin (25 µg mL^−1^). The plates were incubated at 30 °C for 48 h and screened for the presence of transformed colonies. The same protocol was followed for the transformation of *E. coli* DH5α and *S. typhimurium* with a few modifications, where heat shock was given for 90 s followed by incubation at 37 °C for an hour. After incubation, cells were centrifuged and pellets were dissolved in 100 μL of Luria broth (HIMEDIA, M575). The dissolved pellets were spread onto LB agar (HIMEDIA, M557 + agar) plates containing gentamycin (25 µg mL^−1^). The plates were incubated at 37 °C overnight and screened for the presence of transformed colonies.

### Infection of yeast with fluorescent bacteria

The transformed fluorescent *P. stutzeri* JC703, *E. coli* DH5α and *S. typhimurium* as well as auto-fluorescent *Prochlorococcus *sp. and *Rhodopseudomonas palustris* were used for infecting *C. tropicalis* JY101. Cells were first grown in Yeast Carbon Base (YCB) broth (HIMEDIA, M141) in the absence of additional nitrogen sources at room temperature for 24 h. The fluorescently labelled *P. stutzeri* JC703, *E. coli* DH5α, *S. typhimurium* or the auto-fluorescent *Rhodopseudomonas palustris* TIE-1 and *Prochlorococcus* sp. cells were co-cultured with *C. tropicalis* cells in YCB medium devoid of additional nitrogen sources at room temperature. Co-cultures were observed periodically under confocal microscope (Zeiss LSM880) with 63X objective lens. Cells were processed for scanning electron microscopy as described earlier^35^ and the images were taken on Philips XL3O SEM. The co-cultures of *C. tropicalis* and mCherry labelled fluorescent *P. stutzeri* were also analysed by FISH as described above and observed under confocal microscope.

## Results

### Diversity and identity of yeasts

A total of twenty-eight yeasts and one yeast-like fungus were isolated and purified from diverse habitats (Table 1). These were identified based on the D1/D2 domain of large ribosomal subunit or ITS regions (Table 1). Eight strains (JY101, JY106, JY107, JY108, JY113, JY114, JY125, JY134) had the highest (> 99%) identity with *Candida tropicalis.* While other species of the genus *Candida* include; *Candida metapsilosis* (JY103), *Candida suratensis* (JY121, JY124) and *Candida ampae* (JY135). Six strains (JY105, JY112, JY116, JY131, JY129, JY136) showed maximum identity (99%) with the genus *Pichia*. Four strains belonged to the genus *Rhodotorula* (JY109, JY127, JY132, JY143), two strains (JY104 and JY117) to *Meyerozyma*, and a single strain each belonging to the genera *Hanseniaspora* (JY102), *Debaryomyces* (JY130) and *Sporidiobolus* (JY133) (Table 1). The yeast-like fungus with strain number JY119 was identified as *Zalaria obscura*. All the strains were preserved in 50% glycerol at − 20 °C.

### Confirmation of axenic cultures of yeast

As described in methodology, multiple methods were adopted to establish the “axenic” nature of all yeast cultures. This was mainly done by screening all yeast strains using: repeated subculturing on various media, microscopic studies of yeast cells with viability stains, FISH analysis and microscopy**.** These methods demonstrated no external contamination of bacteria.

### Microscopic observation of bacteria like bodies

While observing the morphology of yeast cells under phase-contrast microscope, rapidly moving bodies roughly the size of bacteria were seen in yeast cells (Fig. S1a and Movie S1). Simple bacterial fluorescent staining was done using Texas Red (Fig. S1b) and also the yeast DNA was counterstained with DAPI/SYTOX (Fig. S1c; Movie S2). Twenty-five out of twenty-nine strains of yeast showed such bacteria like bodies (BLBs) in their cells as confirmed from staining. Further, we also observed more than one BLB in the yeast (Movie S2) for which we looked into the bacterial diversity of yeast through metagenome sequencing. FISH was also performed for *C. tropicalis* using rRNA bacteria specific probe EUB338-Cy3.

### Composition and diversity of bacterial communities

Before the extraction of the genomic DNA of yeast, we made sure that there were no external bacteria as observed under the confocal microscope after staining the bacteria. Genomic DNA was extracted from all the twenty-nine strains of yeast and 16S rRNA gene-based metagenome sequencing of the V1–V3 region was performed. Except for the yeast strains JY127, JY132, JY135 and JY143, the remaining twenty-four yeast strains as well as yeast-like fungal strain JY119 yielded good sampling depth as observed from the rarefaction curves (Fig. S2). The same four strains also yielded negative results for staining, suggesting that they may lack bacteria. The metagenome data of the yeasts are deposited with NCBI and the Sequence Read Archive (SRA) accession numbers are given in Table 1. Since *Candida* and *Pichia* were among the largest (represented by about 48% and 24%, respectively) of our collection of yeast in this study, we present the metagenome results of these separately.

### Bacterial community of *Candida* spp

**Figure 1:**
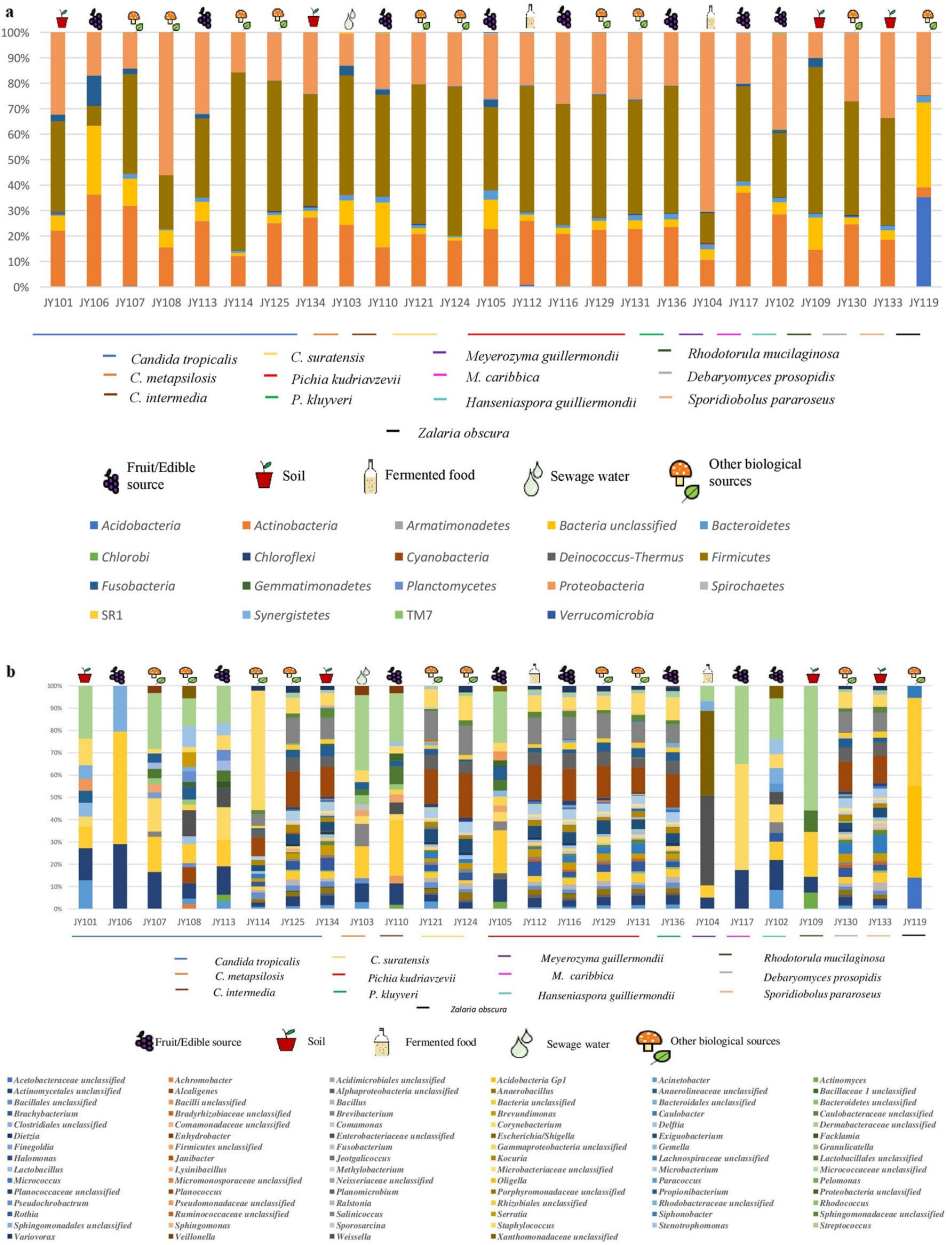
Stacked bar plots showing the relative abundances of bacterial taxa reads distributed among the twenty-five yeast strains; (**a**), taxa at phylum level; (**b**), bacteria at genus level. X-axis indicates yeast strains and Y-axis indicates the relative abundance of bacterial taxa calculated as percentage of Operational Taxonomic Units (OTUs). The plots are supported with legends showing the yeast strains, bacterial taxa as well as environmental sources of yeast isolation. Data on bacterial taxa reads found in each yeast was obtained using mothur V. 1.41.1^24–26^. Plots were constructed using Microsoft Excel and images are self-made using Microsoft power point.

**Figure 2:**
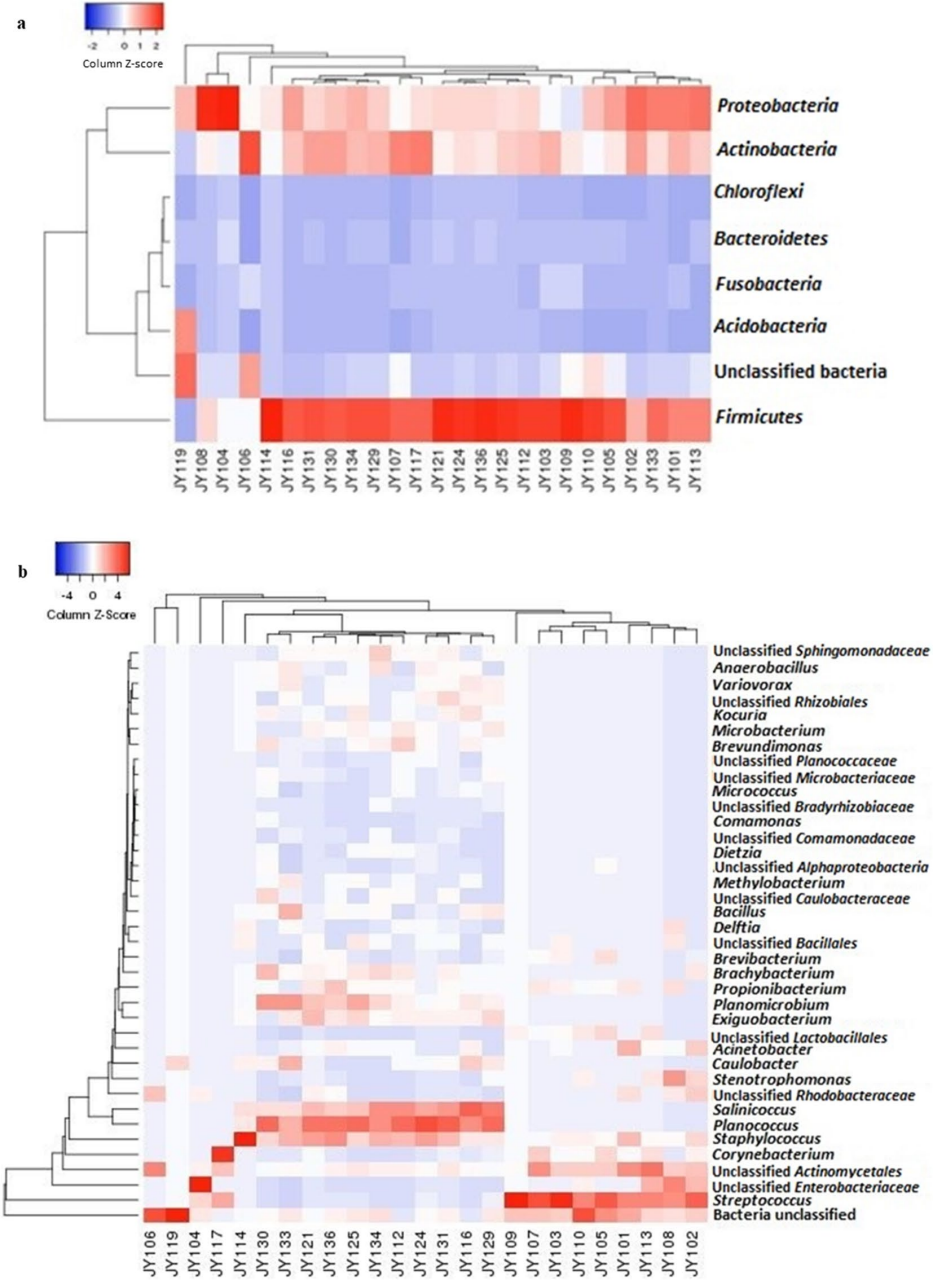
Heatmaps on bacterial abundance in twenty-five yeast strains at; (**a**), phylum level; (**b**), genus level. Other details as in Fig. 1. OTU data was first filtered and normalised using total sum-scaling in MicrobiomeAnalyst web-based tool^27^. The heatmaps were then constructed using Heatmapper web-based tool^29^. Clustering was applied to both column and row data with average linkage method and distances were measured as Euclidean distances. Red colour indicates high abundance and blue colour indicates low abundance as given in the colour scale on the top of the maps.

**Figure 3:**
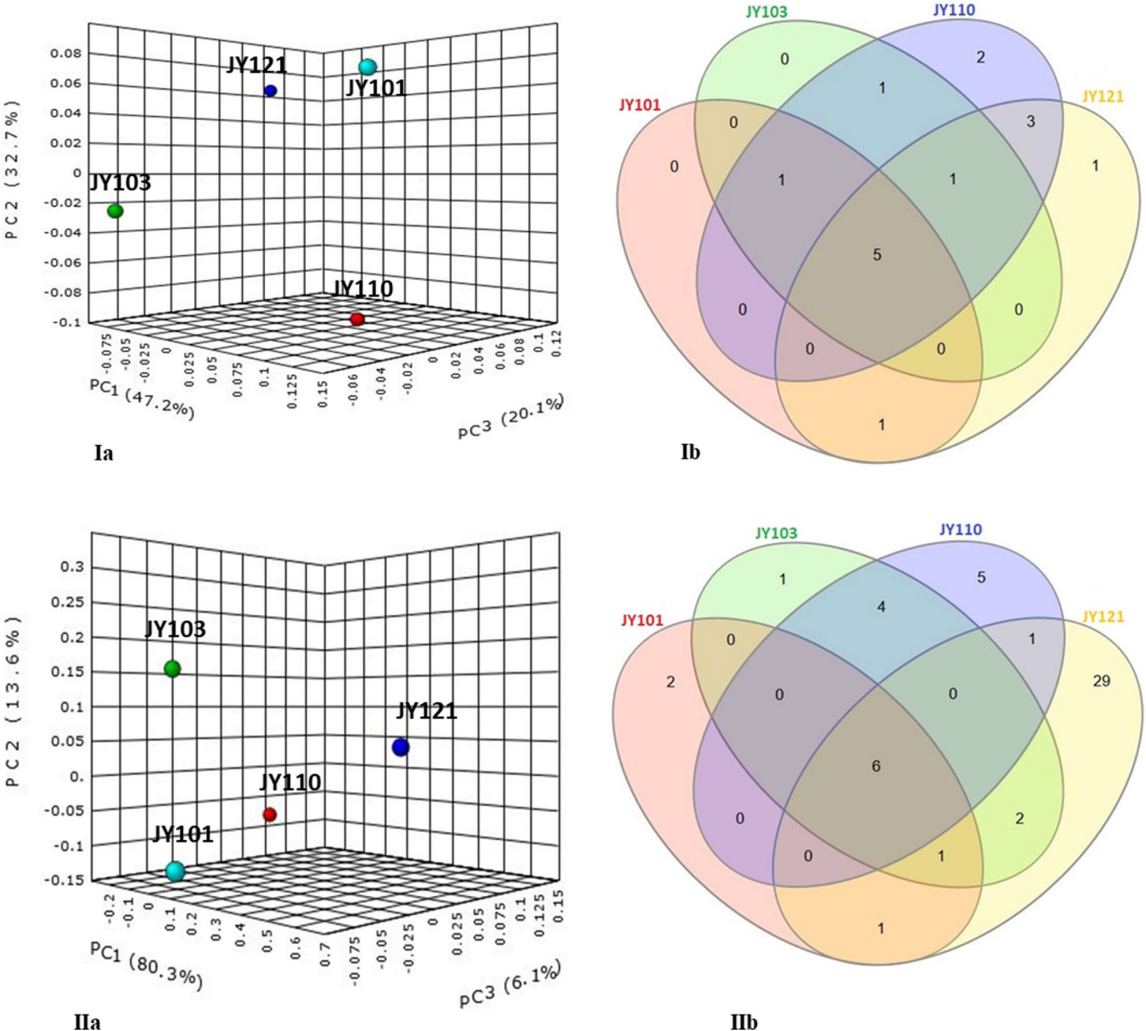
Bacterial diversity among different *Candida* spp. for intra-genera comparison of yeast strains; (**Ia**, **Ib**) PCoA plot and Venn diagram of bacterial phyla distribution in yeast, (**IIa**, **IIb**) PCoA plot and Venn diagram of bacterial genera distribution in yeast. principal coordinates analysis (PCoA) plots were constructed at both phylum and genus level using MicrobiomeAnalyst web-based tool^27^ for the assessment of bacterial beta-diversity between *Candida* strains using Bray–Curtis index. Venn diagram constructed using InteractiVenn web-based tool^28^ to compare common and unique bacterial phyla and genera of the *Candida* strains. Other details as in Fig. 1. Summary of common and unique bacterial taxa derived from Venn diagram is explained in Tables S1 and S2.

The highest number of OTUs (3181) was observed from strain JY121 and lowest number (529) from JY101. *Firmicutes* constituting approximately 49% of the OTUs were the most abundant phylum in all strains of *Candida* (Figs. 1a, 2a, 3Ia). Other reads mapping to *Actinobacteria* (~ 20%), *Bacteroidetes* (~ 2%), *Proteobacteria* (~ 20%) and unclassified bacteria (~ 7%) were common to the four species (Fig. 1a). Further, differences in their bacterial composition are presented in the beta diversity plot using Bray–Curtis index with the permutational MANOVA (PERMANOVA) statistical method. The plot shows (Fig. 3Ia) the individuality of OTUs make-up in each strain, clearly displaying no correlation among them, despite sharing some common trends at the level of phylum (Fig. 3Ia) or genus (Fig. 3IIa). Further comparison at the phylum level among these strains is given in Table S1 and Venn diagram (Fig. 3Ib).

Varied results were observed at the genus level where *Streptococcus* (~ 10%) was common among all the *Candida* spp. with maximum OTUs (210) in strain JY103. *Planococcus* reads were highest in strain JY121 with 446 OTUs. Genera like *Corynebacterium* (~ 2%), *Propionibacterium* (~ 3%), *Staphylococcus* (~ 7%), unclassified bacteria (~ 9%) and unclassified *Actinomycetales* (~ 6%) were also common to all *Candida* spp. (Figs. 1b, 2b, 3IIb). Besides, unclassified *Pseudomonadaceae* reads were unique to strain JY101 having 25 OTUs (Table S2; Fig. 3IIb).

We further compared six strains of *C. tropicalis* (JY101, JY107, JY108, JY113, JY114 and JY125) for intra-species analysis of bacterial diversity. Yeast strain JY125 had the greatest number of OTUs (3125) and strain JY101 had the least (529). *Deinococcus-Thermus*, *Spirochaetes*, *Gemmatimonadetes* were the unique phyla of bacterial diversity of *C. tropicalis* strains JY101, JY107 and JY114 respectively (Fig. S3Ia,Ib; Table S3). *Firmicutes* constituted approximately half the total read share (up to 46%) in these strains. *Actinobacteria* (~ 22%) and *Proteobacteria* (~ 23%) were the next abundant phyla with a similar number of reads. Genus level analysis (Fig. S3IIa,IIb; Table S4) showed the presence of the genus *Staphylococcus*, unclassified bacteria and unclassified *Actinomycetales* in all the *C*. *tropicalis* strains. Although diversity was more varied at the genus-level, few taxa showed high read dominance. For example, *Staphylococcus* (~ 54%) was abundant in strain JY114 while *Streptococcus* and *Planococcus* reads were in similar proportions in the rest of the *C. tropicalis* strains. As mentioned previously, unclassified *Pseudomonadaceae* reads only showed up in strain JY101.

### Bacterial community of *Pichia* spp

**Figure 4:**
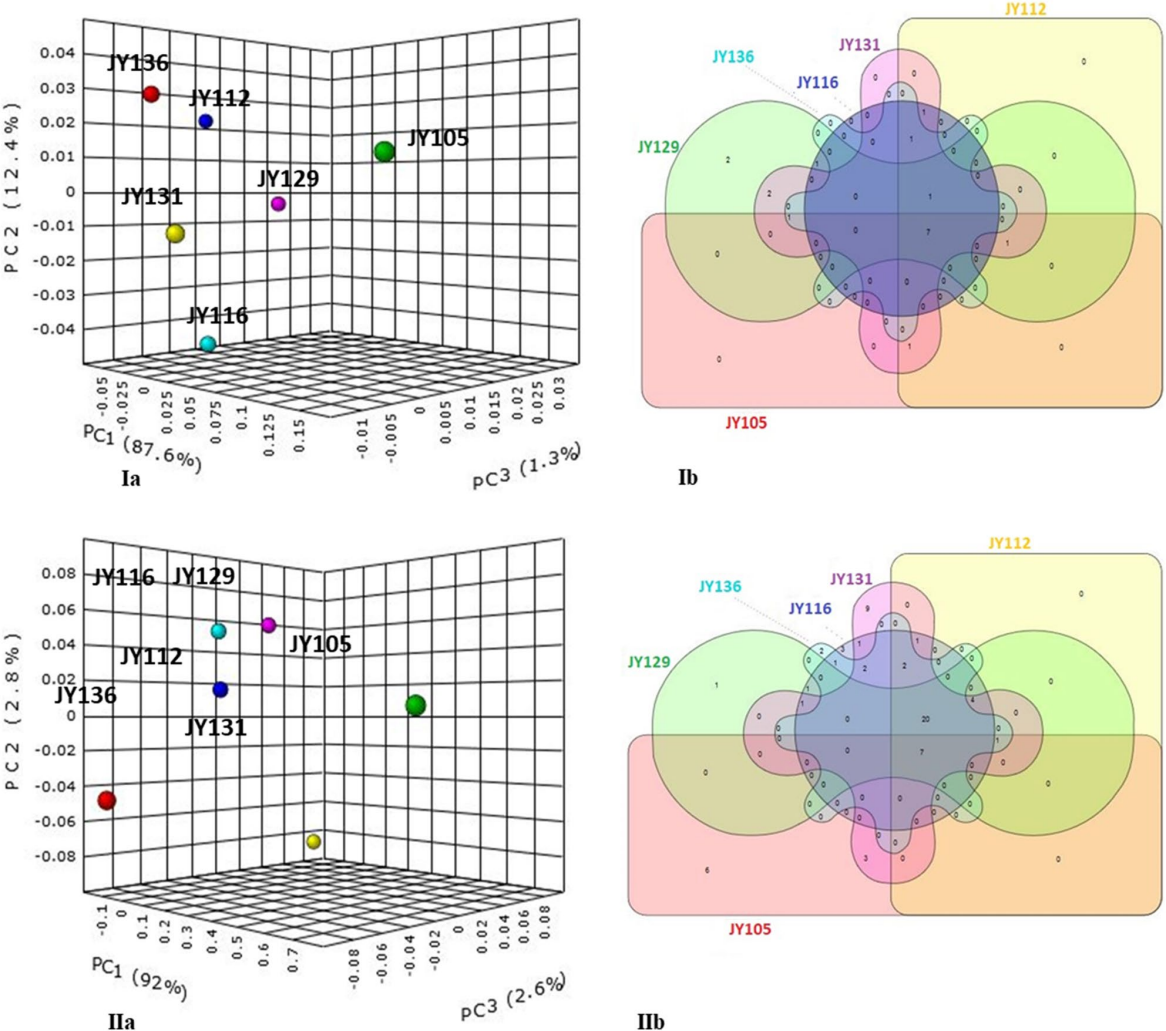
Bacterial diversity among different *Pichia sp* for intra-genera comparison of yeast strains; (**Ia**, **Ib**) PCoA plot and Venn diagram of bacterial phyla distribution in yeast, (**IIa**, **IIb**) PCoA plot and Venn diagram of bacterial genera distribution in yeast. Other details in Fig. 3. Summary of common and unique bacterial taxa derived from Venn diagram is explained in Tables S3 and S4.

Bacterial reads of *P. kudriavzevii* (JY105, JY112, JY116, JY129 and JY131) and *P. kluyveri* (JY136) were compared similarly at bacterial phylum and genus level. Strain JY131 had maximum number of OTUs of 5524 and strain JY105 had minimum number of 1015. Diversity measures for *Pichia* spp. (Fig. 4Ia,Ib) were calculated as done for *Candida* spp. *Firmicutes* constituted 46% and was the most abundant of the reads among all *Pichia* strains. *Acidobacteria* (~ 0.3%), *Actinobacteria* (~ 23%), *Bacteroidetes* (~ 2%), *Firmicutes* (~ 46%), *Planctomycetes* (~ 0.2%), *Proteobacteria* (~ 25%) and unclassified bacteria (~ 3%) were the common phyla for all strains (Fig. 3Ia; Table S5). Whereas *Chlorobi* and *Deinococcus-Thermus* were found to be specific for strain JY129 (Fig. 4IIa,IIb; Table S5).

At genus level, *Staphylococcus* (~ 8%), *Propionibacterium* (~ 3%), *Streptococcus* (~ 2%) and *Corynebacterium* (~ 1%) OTUs were common in the total read share. Genus *Planococcus* was present in all strains except JY105, showing high read dominance and contributing a total of 13% of OTU count (Fig. 4IIa,IIb). Reads of *Weissella* were only found in yeast strain JY136. Reads of unclassified *Actinomycetales*, *Granulicatella*, *Gemella,* unclassified *Lactobacillales* and unclassified *Pseudomonadaceae* were present only in yeast strain JY105 (Table S6). Unclassified *Micromonosporaceae* OTUs were uniquely present in strain JY129. Yeast strain JY131 had the OTUs of genus *Siphonobacter*. Further diversity analysis of different strains of the genus *Pichia* are given in Table S6 and Venn diagram (Fig. 4IIb).

### Diversity indices of all yeast strains

**Figure 5:**
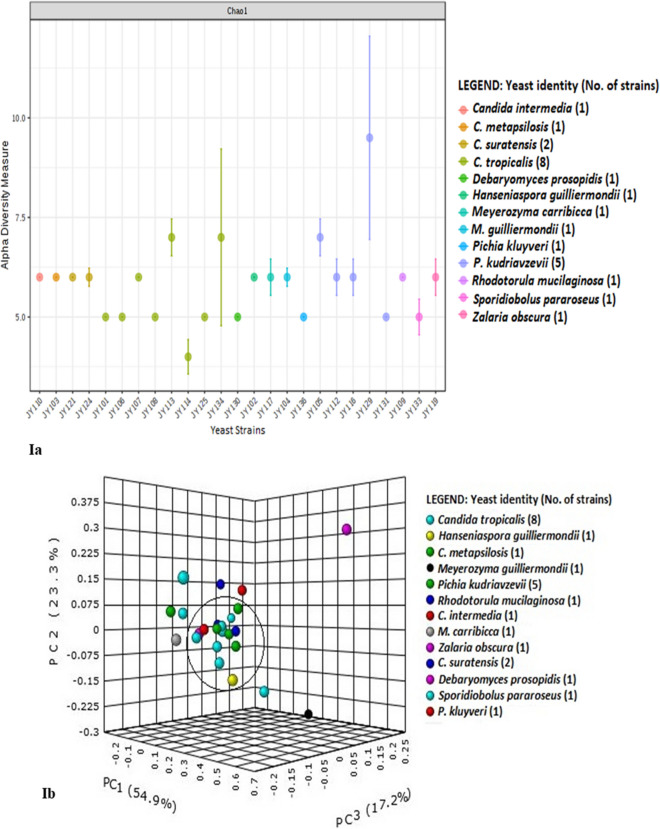

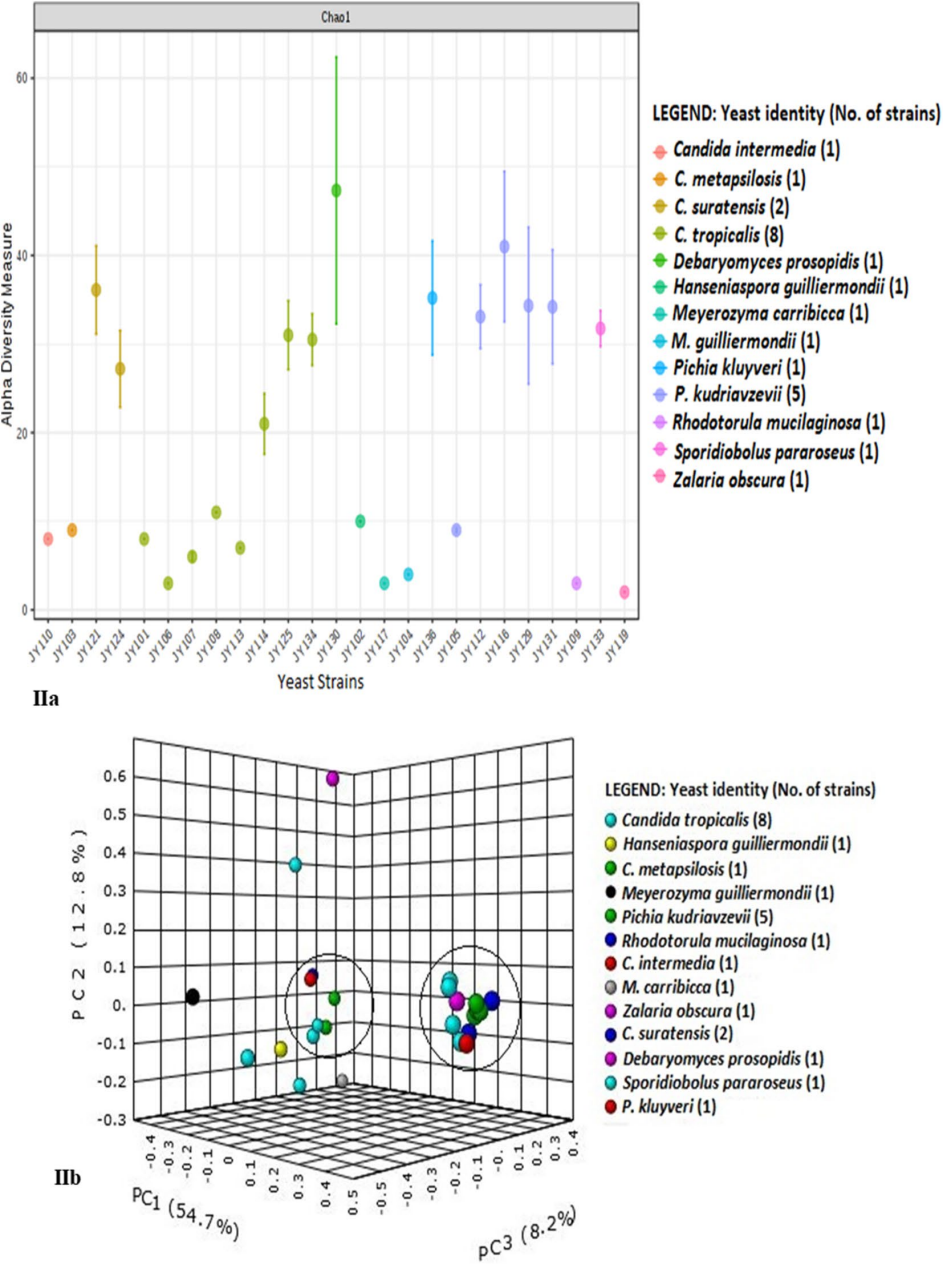
Bacterial diversity among all twenty-five yeast strains; (**Ia**, **Ib**) Alpha and beta diversity of bacterial phyla distribution in all yeast, (**IIa**, **IIb**) Alpha and beta diversity of bacterial genera distribution in all yeast. Alpha diversity was measured by Chao-1 index and beta diversity with PCoA plots was measured with Bray–Curtis index at bacterial genus and phylum levels for all yeast strains. Other details as in Figs. 3 and 4. Summary of common and unique bacterial taxa derived from Venn diagram is explained in Tables S5 and S6.

Comparative metagenome diversity of all the 25 yeasts showed bacterial read heterogeneity among all the yeast as analysed by diversity indices (Fig. 5), bar plots (Fig. 1a,b) and heatmaps (Fig. 2a,b). The maximum number (5539) of OTUs was seen for *P. kudriavzevii* JY131 whereas the least (218 reads) was found in *C. tropicalis* JY106. Alpha diversity using the Chao-1 index and T-test/ANOVA statistical method (Fig. 5Ia,IIa) and beta diversity using Bray–Curtis index and PERMANOVA statistical method were examined (Fig. 5Ib,IIb) to validate our diversity analysis. Several unclassified reads were also detected at various taxonomic levels, with unclassified bacterial reads at phylum level contributing to significant abundance in strains JY106 (27%), JY109 (13%), JY110 (18%) and JY119 (33%) which would either need further analysis or corroboration.

*Firmicutes* (~ 45%) remain the dominant phylum followed by *Proteobacteria* (~ 25%) and *Actinobacteria* (~ 22%) for all the yeasts examined in this study (Figs. 1a, 2a). At the genus level, major differences in the abundance were observed (Figs. 1b, 2b). Only a few bacterial genera were common among certain groups of yeast strains, as shown by the clustering of these samples in the PCoA plots (Fig. 5IIb). Reads of the genus *Streptococcus* contributed the greatest number of OTUs in all yeast strains, with the maximum number in *C. metapsilosis* strain JY103 (~ 34% of their total OTUs) and the minimum in *C. suratensis* strain JY121 (~ 4% of their total). Strain JY119 was the only strain that did not possess any *Streptococcus* reads. It had notably different read composition from the rest of the yeast strains where *Acidobacteria* (236 OTUs) was the most dominant phylum. Also, JY119 showed lesser richness of bacterial diversity as against the majority of the yeast strains. *Planococcus* reads dominated in strains JY112, JY116, JY121, JY124, JY125, JY129, JY130, JY131, JY133, JY134 and JY136 with the maximum number of OTUs in *Candida suratensis* strain JY124 (~ 20% of their total) and the minimum in *P. kudriavzevii* strain JY131 (~ 11% of their total). OTUs of unclassified *Clostridiales* were only observed in *P. kudriavzevii* JY131. Diversity and abundance of the bacterial community of the yeasts have been represented in the form of bar graph and heat map at phylum as well as at genus level (Figs. 1a,b, 2a,b).

### Insights into bacteria associated with *Candida tropicalis* strain JY101

We selected *C. tropicalis* strain JY101 as a model yeast for further studies to validate the bacterial diversity. This was done through FISH studies followed by cultivating the bacteria from the yeast and re-infecting the yeast strain using a fluorescently labelled bacterium to mimic Koch’s postulates.

### FISH studies

**Figure 6:**
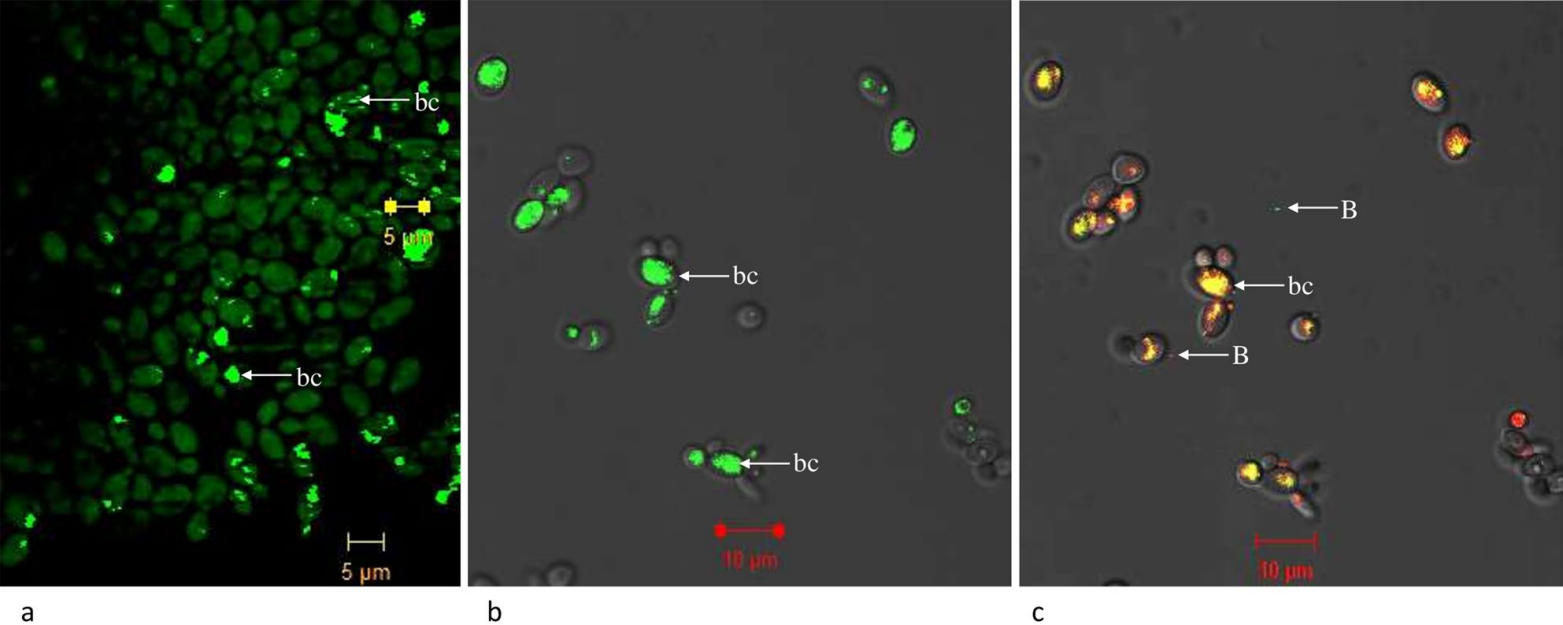
Confocal fluorescence microscopic FISH images of *Candida tropicalis.* (**a**) Native bacterial community (bc) of the yeast showing the green fluorescence inside the yeast cells (**b**) Infection studies showing native bacterial community (bc) of the yeast showing green fluorescent while the mCherry tagged *P. stutzeri* have red fluorescent and an over lay of the image (**c**) shows the presence of both native community along with the infected cells inside the yeast cells. Bacterial communities associated with the yeast cells were stained with rRNA bacterial EUB338-Cy3 probe (green). Arrows indicate: bc, bacterial communities; B, Bacterial cells which are seen outside the yeast.

To corroborate the presence of bacterial communities in yeast, FISH was performed with *C. tropicalis* using Cy3 labelled bacterial specific probe, EUB338. Positive fluorescence observed (Fig. 6a) confirms the presence of endo-bacteria of *C. tropicalis*. We rule out the possibility of positive fluorescence by yeast mitochondria since isolated mitochondria did not show any positive fluorescence using the above probe (data not shown).

### Cultivated and characterized bacteria from yeast

16S rRNA gene metagenome of *C. tropicalis* JY101 showed reads of unclassified *Pseudomonadaceae*, members of which can be cultured easily. Cells of strain JY101 were ruptured with physical (glass beads) and enzymatic (zymolase) treatments. When plated on nutrient broth, this yielded four different bacteria which were purified on nutrient agar (HIMEDIA, M002) and sequenced for 16S rRNA gene-based identification. The bacteria identified were members of genus *Pseudomonas*, *Chryseobacterium*, *Lysinibacillus* and *Propionibacterium*. EzTaxon BLAST search of the 16S rRNA gene sequence (1387 nt) of *Pseudomonas* strain JC703 had the highest (99.9%) identity with *Pseudomonas stutzeri* ATCC 17588^T^ followed by other members of the genus *Pseudomonas* with < 99% sequence identity. While the *Chryseobacterium* strain JC507 sequence (1428 nt) had highest (98.7%) identity with *Chryseobacterium indologenes* NBRC 14944^T^ followed by other members of the genus *Chryseobacterium* with < 98.6% identity. *Lysinibacillus* strain JC1018 sequence (889 nt) had the highest (100%) identity with *Lysinibacillus fusiformis* NBRC 15717^T^ followed by < 98.7% with other members. *Propionibacterium* strain JC704 sequence (1396 nt) had the highest similarity (98.6%) with *Propionibacterium acne* DSM 1897^T^ followed by < 95.7% with other members. The 16S rRNA gene sequences of the four bacteria of *C. tropicalis* JY101 were deposited with NCBI with accession numbers LR735276, LT838865, LR735277 and LR743671 respectively for bacterial strains JC703, JC507, JC1018 and JC704. All the cultures have been preserved as glycerol (50% v/v) stocks stored at − 20 °C and *Chryseobacterium* sp. JC507 was deposited with KCTC (KCTC 52928) and NBRC (NBRC 113872) as *Chryseobacterium candidae* JC507^T^. Our attempts failed to cultivate other bacteria from *C. tropicalis* JY101 where we used Marine agar media (HIMEDIA, M384), Mueller Hinton agar (HIMEDIA, M173) and *Thiobacillus* agar media (HIMEDIA, M788).

### Infection studies of* C. tropicalis* JY101

**Figure 7:**
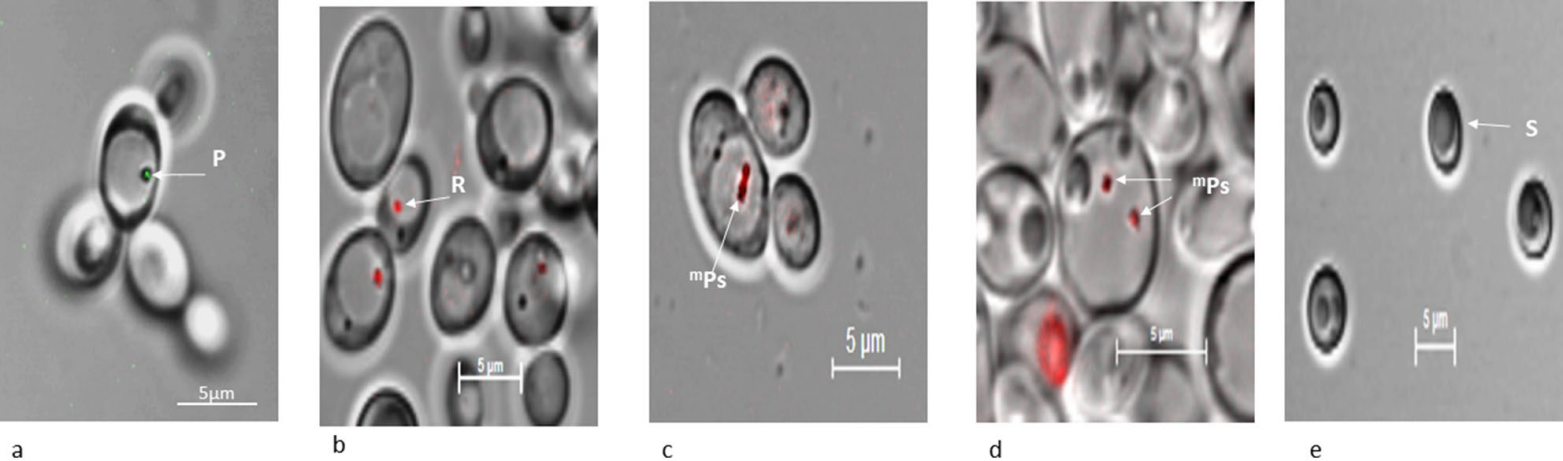
Confocal microscopy images of yeast-bacteria co-cultures under nitrogen deficit showing (**a**) *Prochlorococcus* sp. as green fluorescent bacteria in *C. tropicalis*, (**b**) *Rhodopseudomonas palustris* strain TIE-1 as red fluorescent bacteria in *C. tropicalis*, (**c**,**d**) mCherry tagged *P. stutzeri* as red fluorescent bacteria in *C. tropicalis*, (**e**) Control *C. tropicalis* cells grown in nitrogen deficiency without bacteria*.* Images were magnified with total magnification of 630X with appropriate scale. Arrows indicate: P, *Prochlorococcus*; R, *Rhodopseudomonas*; ^m^Ps, mCherry *Pseudomonas*; S, Spore.

**Figure 8:**
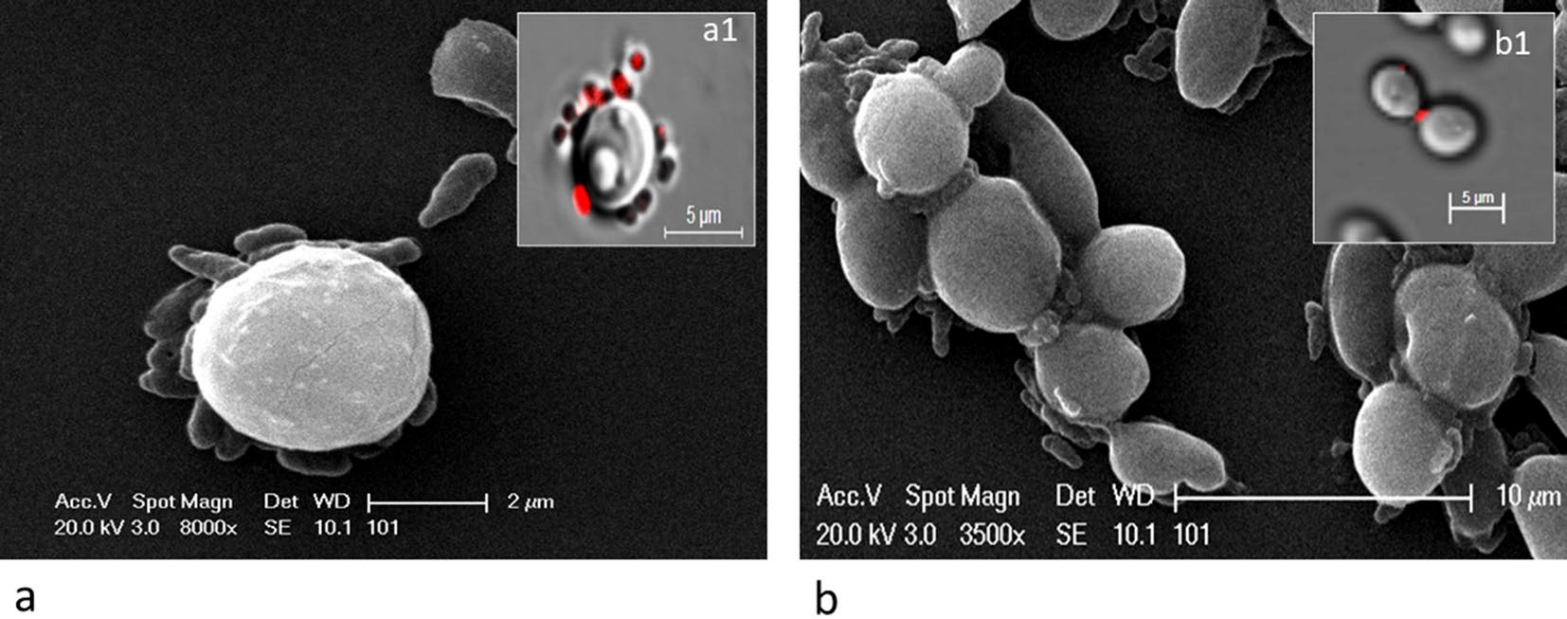
Scanning electron microscopy (SEM) images showing (**a**) *P. stutzeri* bacteria attachments on a *C. tropicalis* cells, (**b**) *P. stutzeri* attachments onto *C. tropicalis* bud collars. Insert: (a1) Confocal microscope image showing similar *P. stutzeri* attachment on a *C. tropicalis* cell, (b1) Confocal microscope image showing similar *P. stutzeri* attachment onto budding *C. tropicalis* cells.

For infection studies, we used auto-fluorescent bacteria *Prochlorococcus* sp. and *Rhodopseudomonas palustris* TIE-1 and mCherry labelled plasmid transformed in *P. stutzeri* JC703, *E. coli* DH5α and *S. typhimurium*. The mCherry tagged *E. coli* DH5α or *Salmonella* did not infect *C. tropicalis* JY101 nor were observed in the yeast whereas the other bacteria yielded successful infection and entry into the yeast in nitrogen deficient media. In *C. tropicalis* JY101, *Prochlorococcus* sp. was observed as green fluorescent bacteria (Fig. 7a), *Rhodopseudomonas palustris* TIE-1 cells were seen as red fluorescent bacteria (Fig. 7b) and mCherry labelled *P. stutzeri* JC703 was observed as red fluorescent bacteria (Fig. 7c,d). The control yeast cells exhibited spore like structures (Fig. 7e) that were not seen in the case of infected yeast cells. Scanning Electron Microscopy (SEM) of infected *C. tropicalis* with *P. stutzeri* helped to further observe the yeast and bacteria (Fig. 8). Bacteria were first seen to adhere to the yeast cell in many numbers (Fig. 8a; counter supported by confocal image Fig. 8a1); particularly at the collar of the budding yeast (Fig. 8b; counter supported by confocal image Fig. 8b1) and were seen inside the yeast cell (Fig. 7d,e; Movie S3). FISH analysis of the *C. tropicalis* along with the infected mCherry tagged *P. stutzeri* showed positive signals on hybridization. Along with the native bacterial community of the yeast (Fig. 6b) which imparts green fluorescence when bind with bacterial rRNA probe EUB338-Cy3 (Fig. 6b), red fluorescence was also observed which was due to mcherry tagged *P. stutzeri*. An overlay image (Fig. 6c) confirms the presence of inherent bacterial community and the infected cells of *P. stutzeri* inside the yeast (Fig. S4).

## Discussion

Endosymbiotic bacteria play an important role in the evolution of higher life forms^35–37^. In this process, some of the endosymbiotic bacteria became integral components of the host as organelles, while a few remained as endosymbionts of fungi^38^, plants^39^ and animals^40^. Endosymbionts have been extensively studied from an evolutionary point of view^41^. To this end, recent studies also tried to establish the endosymbiotic theory of evolution of mitochondria using engineered *E. coli* into *Saccharomyces cerevisiae*^20,21^. While these are artificially generated endosymbionts, naturally harboured “endobacteria” of yeast have only now come under investigation^12–19^, implicating these bacteria in a variety of roles. Our study aimed to extend the understanding of co-occurrence of yeast and bacteria, by first examining the diversity through 16S rRNA gene metagenome analysis and then with infection assays of yeast with fluorescent bacteria.

Yeast cultures were comprehensively subjected to various methods to ensure absence of external bacteria or any other contamination. Initially, BLBs moving inside the yeast were examined for viability using ViaGram staining kit that determines both viability and bacterial nature. This showed that the bodies are viable and could potentially have bacterial origin (Fig. S1). We performed FISH, which is a powerful tool to detect bacteria directly, without the need for cultivation. The rRNA bacterial probe EUB338-Cy3 has been routinely employed and demonstrated to have sufficient power to detect most bacteria. FISH analysis with this probe revealed successful hybridization of the bacteria inside the cells of *C. tropicalis* (Fig. 6a). Repeated streaking and/or subculturing of yeast strains on multiple growth media did not yield any visible bacterial colonies (data not shown). Taken together, these evidences provide a strong case for the hypothesis that these BLBs are indeed bacteria associated within the yeast. With these leads, we analysed the bacterial communities with both culture independent and dependent techniques.

The 16S rRNA gene metagenome analysis showed that about 85% of the yeasts examined in possess bacterial reads and also the presence of the rich diversity of bacteria associated with yeast. Isolation of yeast-like fungus, i.e., *Zalaria obscura* JY119 presented us with an opportunity to utilize its system as an out-group in our study of yeasts. It was intriguing to note that the bacterial diversity of JY119 was markedly different from the rest of the yeast as can be seen from the bar plots and heatmaps. This evidently conveys the specificity of bacterial diversity among fungal organisms. We analysed yeast genera-specific diversity patterns (Figs. 1b, 2b, 5IIa,IIb) and those spanning among all the strains (Table 1). Overall, taxa of the phylum *Firmicutes* dominated in all the yeasts analysed in this study. This may be attributed to their ubiquitous nature and the existence of its numerous spherical cell shaped members because the dominant Firmicutes taxa of yeasts were *Streptococcus, Staphylococcus* and *Planococcus* (Figs. 1, 2)*.* In correlation with these data, the moving BLBs first seen in yeast cells were also coccoidal. This could point to the significance of the shape of the bacteria present inside yeast cells. Also, several reads of rod-shaped bacteria notably from unclassified *Pseudomonadaceae* family were obtained. Possible heterogeneity in the morphology of these bacteria cannot simply be explained (in this case) through phase contrast microscopic studies (Fig. S1a) or from FISH studies (Fig. 6a). However, the morphological distinction in cell shape and size can be clearly seen in Movie S2 where, if closely observed, two distinct morphological structures i.e., one stained with green is distinct from another BLB stained red.

Often metagenome results on microbiota are linked to the properties of the habitat from where DNA is isolated. However, from our results, the bacterial diversity did not show any dependence on environmental conditions from which the yeast hosts were isolated (Fig. 1). Instead, the diversity was specific to the yeast host itself, as indicated by the comparison among *Candida* and *Pichia* species (Figs. 3, 4, S3). However, without more experimentation, these correlations are only at the best, suggestive. This culture-independent method enabled us to explore the existence of a bacterial “community” as against studies reporting the presence of single bacterial species inside the yeast cell^12–17^. Also, as many as 10^7^ live bacteria were counted in a single spore of the Arbuscular Mycorrhizal Fungus, *Gigaspora margarita*^42^ which supports the existence of communities. Uncovering a wealth of bacterial reads from twenty-five different yeasts in this study reveals substantial numbers of bacteria that cannot be easily dismissed. Consequently, future studies should focus on understanding where and how the yeast hosts these bacteria.

Further, the data was validated to an extent by our successful attempts to isolate some of these bacteria from *C. tropicalis* JY101 by culture-dependent methods. Out of the four isolated bacteria, three (*Pseudomonas stutzeri* JC703, *Lysinibacillus fusiformis* JC1018, *Chryseobacterium* sp. JC507) were successfully sub-cultured and preserved, while *Propionibacterium acnes* JC704 (renamed as *Cutibacterium acnes*; Ref.^43^) lost its viability soon after its isolation. It has been demonstrated that many obligate fungal endosymbionts such as “*Candidatus* Glomeribacter gigasporarum” are uncultivable^44^. Perhaps, *Propionibacterium acnes* has become obligately associated with yeast maybe by the loss of some essential genes making it dependent on the host. The reads of *Propionibacterium acnes* were common to all the 25 yeast strains that again begs the question of its obligate relationship. Interestingly, *P. acnes* has been observed to adapt to new organisms as an endosymbiont, displaying flexibility for hosts^45^. Therefore, it may not be unusual to note the presence of *Propionibacterium* along with yeast cells. Among those that were sub-cultured, *Chryseobacterium* sp. JC507 was described as a new species and named as *Chryseobacterium candidae*^13^. Out of 12 genera identified through the metagenome of *C. tropicalis*, only members of three genera could be successfully cultivated in this study. Hence, a large number (~ 95%) remains yet-to-be cultivated or lost cultivability, indicating an unexplored black box of diverse microbiota of yeast.

The function and role of these bacteria of yeast are still to be elucidated, particularly concerning the host which bears the cost of harbouring them (Fig. 6a). The recent finding on interactions between the yeast *Rhodotorula mucilaginosa* harbouring N_2_-fixing *P. stutzeri* and rice plants highlights the role of endosymbiotic bacteria in the nitrogen needs of plants^18^. This example parallels the role of endobacteria of plant-associated filamentous fungi^19^. Similarly, certain species of the isolated bacteria from our study as well as *Rhodopseudomonas palustris* used for infection assays, are known to harbour *nif* genes that help in nitrogen fixation^46,47^. We hypothesize that this capability could be of use to the yeast host and potentially form a bipartite system. We started with a simple strategy to infect a yeast host with the isolated bacteria. Co-cultures of *C. tropicalis* JY101 along with fluorescently labelled *P. stutzeri* JC703 showed the establishment of *P. stutzeri* JC703 inside the yeast cell under nitrogen crisis. The control cultures of *C. tropicalis* JY101 without *P. stutzeri* formed spore like structures with poor growth (Fig. 7e). Devoid of a helpful partner, the control yeast cells possibly coped in the absence of an important nutrient like nitrogen by forming spores. These observations support our hypothesis that *P. stutzeri* and other bacteria aid the host under nitrogen deprivation, providing scope for further analysis. Although no reads of *Prochlorococcus* sp. were observed in the yeast metagenome, we have seen its entry into the yeast under nutrition deficiency (Fig. 7a). We speculate that this demonstrates the ability of the yeast to acquire bacteria, if the situation demands. In such cases, these bacteria could be temporary residents where they may be considered as beneficial guests to the yeast under stress. This is unlike the case where bacteria like *Rickettsia* that have, through obligate endosymbiosis, evolved into organelles like mitochondria. These interesting findings of infection should be carefully explained with supporting experimental designs that can unravel the molecular events controlling this phenomenon and critically probe the process of bacterial infection of yeast. At this stage comparison of the massive diversity from metagenome with the infection of one or two bacteria of the yeast still raises a question on this anomaly.

Our study for the first time shows the existence of large bacteria communities in yeast. Collectively, present and future studies will provide insights into the ecology and evolution of the bond between a host and its endosymbiont. Genome size reduction of *E. coli* engineered as a yeast endosymbiont^21^ describes a crucial stage in the evolution of eukaryotic organelles. This type of event must be looked for in natural yeast-bacterial systems as well, though we argue that we cannot yet firmly categorize which of these bacteria are permanent or transient or whether they are indeed obligate endobacteria. Moreover, we realise that the current evidences from this and previous studies fall short in addressing the function of yeast associated bacteria as our work aimed to provide a preliminary examination into vexing conundrum of ghost bacterial communities of yeast.

## Supplementary information

Supplementary Video 1.

Supplementary Video 2.

Supplementary Video 3.

Supplementary Information 1.

Supplementary Information 2.

## Data Availability

Raw FastQ files of the 16S rRNA metagenomes of all 25 yeast strains were deposited in National Center for Biotechnology Information Sequence Read Archive and are available in NCBI SRA under accession numbers given in Table 1.
