# Supplementary material for: Uncovering the hidden bacterial ghost communities of yeast and experimental evidences demonstrates yeast as thriving hub for bacteria
Source: Sci Rep. 2021 Apr 30;11:9394. doi: 10.1038/s41598-021-88658-x (PMC8087679; doi:10.1038/s41598-021-88658-x)
Supplement: Supplementary file 4 — Supplementary Information 1. [file 41598_2021_88658_MOESM4_ESM.pdf]

Uncovering the hidden bacterial ghost communities of yeast and experimental evidences demonstrates yeast as thriving hub for bacteria

B Indu[1#], Tallapragada Keertana[1#], Sahu Ipsita[1], Uppada Jagadeeshwari[2], Chintalapati Sasikala[2] and Chintalapati Venkata Ramana*[1]

[1]Department of Plant Sciences, School of Life Sciences, University of Hyderabad, Hyderabad, India.

[2]Bacterial Discovery Laboratory, Centre for Environment, Institute of Science and Technology, J.N.T. University Hyderabad, India.

#both the authors contributed equally

***Author for correspondence: Ch. V. Ramana**

e-mail: cvr449@gmail.com; chvrsl@uohyd.ernet.in

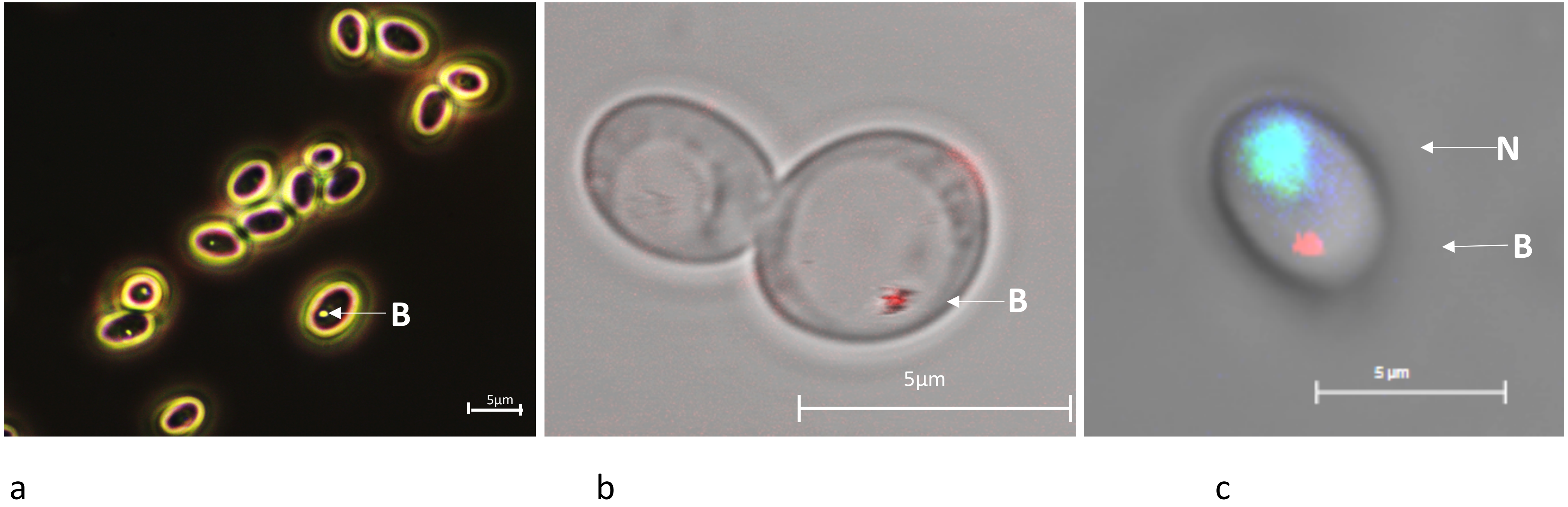

Fig S1. Microscopic examination of yeast cells.

a, Phase contrast microscopy of *C. tropicalis* JY101 showing the presence of bacteria inside the yeast; b, Confocal microscopy of *C. tropicalis* JY101 with bacteria when stained for viability using Texas Red; c, Confocal microscopy of *C. tropicalis* JY101 with bacteria when stained for viability using DAPI, SYTOX and Texas Red. Staining was done with ViaGram viability staining kit. Arrows: B, Bacteria like bodies; N, Yeast nucleus.

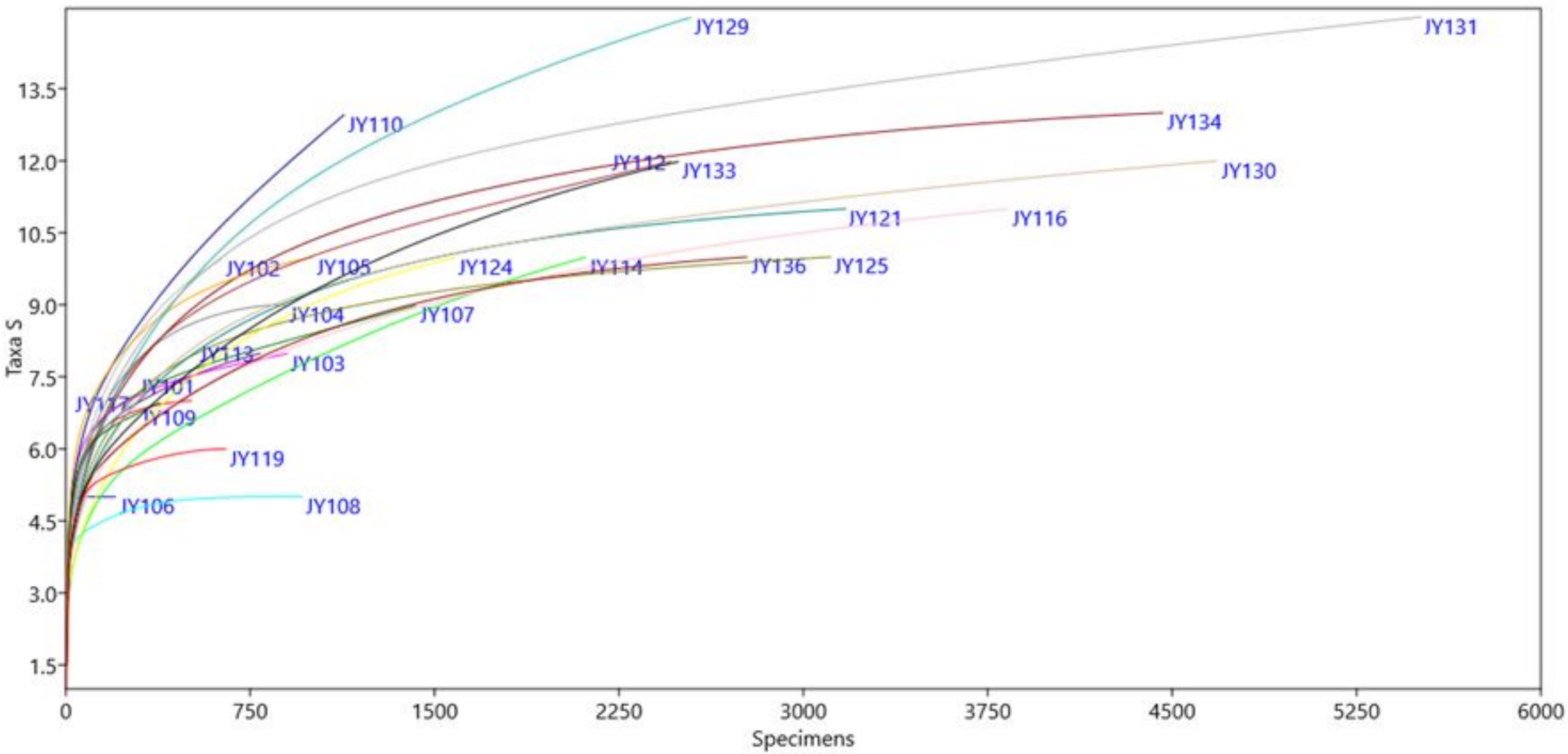

Fig S2. Rarefaction curves of all strains for their V1-V3 region of 16S rRNA gene metagenomes showing the saturation of bacterial diversity and richness of each yeast strain.

Data on bacterial taxa reads found with each yeast strain was obtained using mothur V. 1.41.1 [24] and the curves were constructed using PAST V3.26 [30].

Fig S3. Bacterial diversity among six *Candida tropicalis strains* showing intra-species comparison.

Ia, Ib: PCoA plot and Venn diagram of bacteria at phyla distribution.

IIa, Iib: PCoA plot and Venn diagram of bacteria at genus distribution.

Principal Coordinates Analysis (PCoA) plots were constructed at both
phylum and genus level using MicrobiomeAnalyst web-based tool [27] for the assessment of bacterial beta-diversity between *C. tropicalis* strains using Bray-Curtis index. Venn diagram constructed using InteractiVenn web-based tool [28] to compare common and unique bacterial phyla and genera of the *C. tropicalis* strains. Data on bacterial taxa reads found in each yeast was obtained using mothur V. 1.41.1. [24].

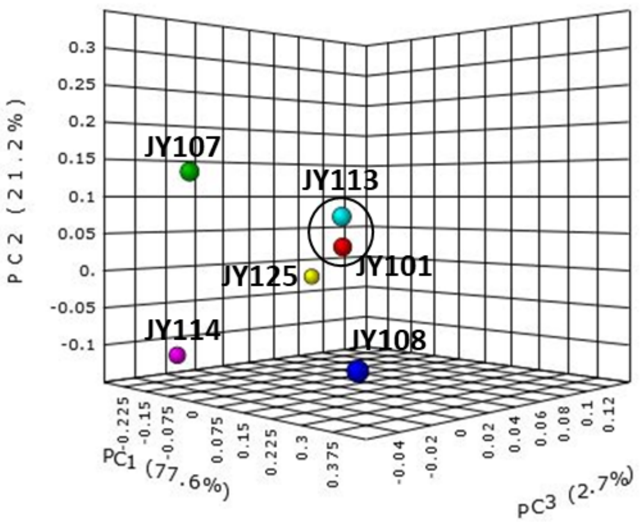

Ia

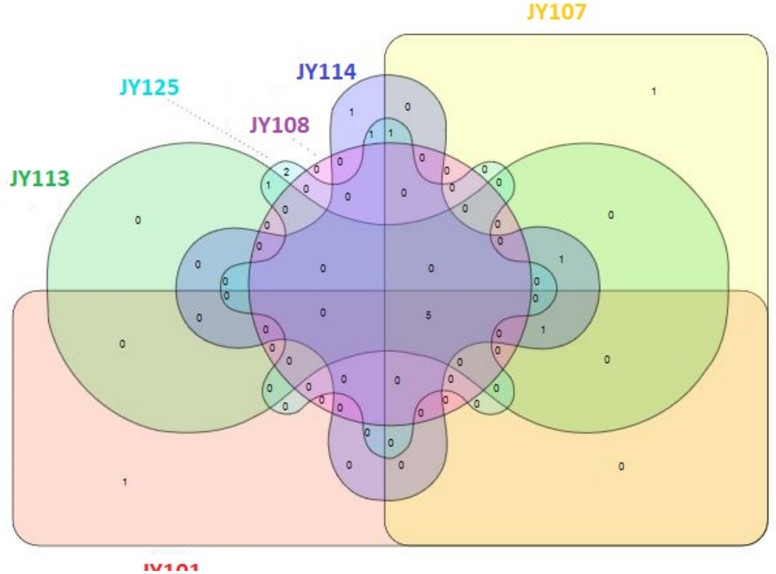

Ib

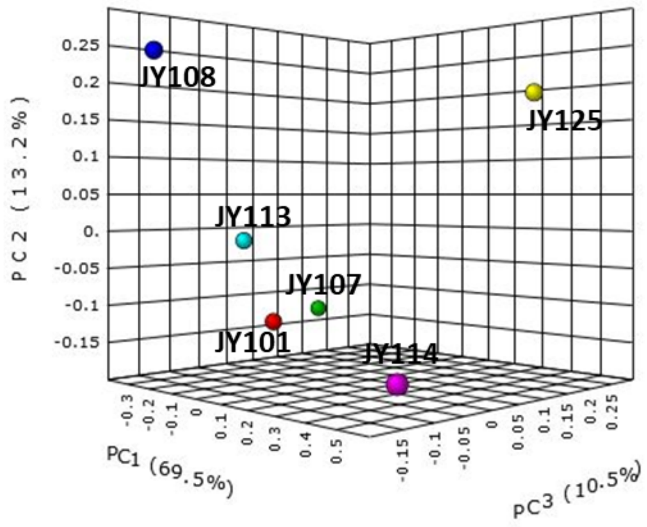

IIa

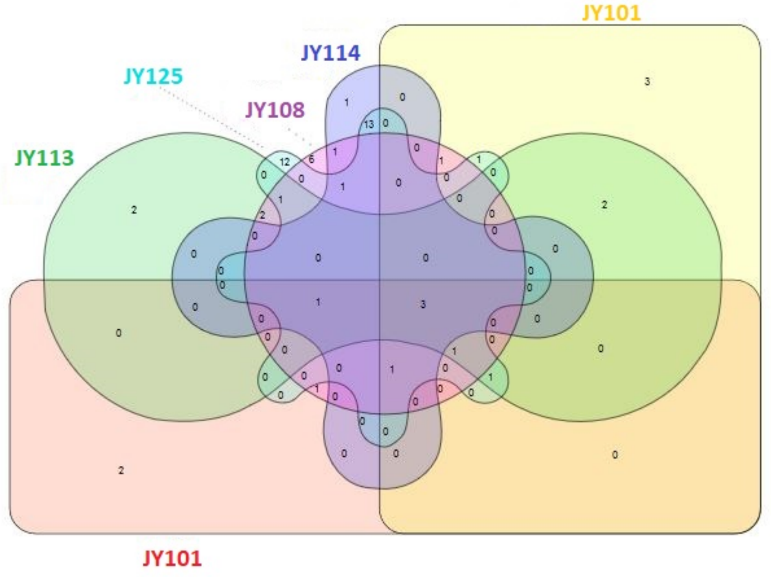

IIb

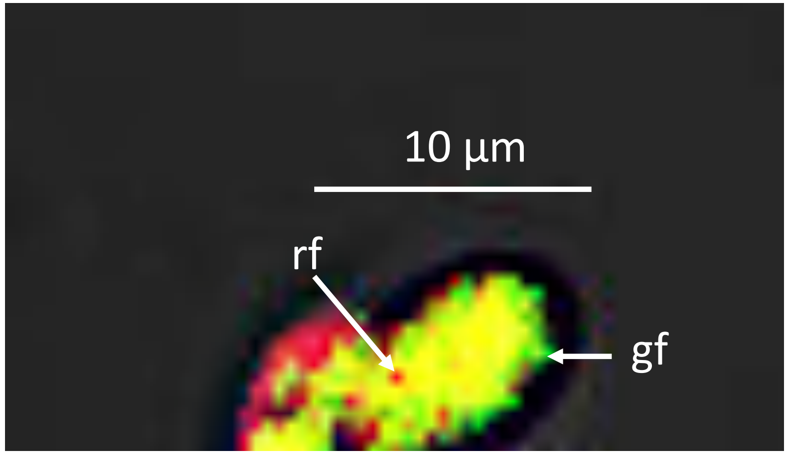

Fig S4. Confocal microscopic image of *C. tropicalis* cells showing Cy3 hybridized cells of bacteria with green fluorescence while the mCherry tagged *P. stutzeri* infected bacterial cells with red fluorescence. Arrow indicate: rc, red fluorescence (rf) *P. stutzeri* cells; gc, green fluorescence (gf) bacterial cells

Table S1. Common and unique bacterial taxa at phylum level between *Candida* spp.

Yeast Strain(s)	Bacterial Phyla
JY110	*Armatimonadetes*, TM7
JY121	*Acidobacteria*
JY101, JY103 & JY110	*Fusobacteria*
JY101, JY103, JY110 & JY121	*Actinobacteria*, Unclassified bacteria, *Bacteroidetes*, *Firmicutes*, *Proteobacteria*
JY103 & JY110	SR1
JY103, JY110 & JY121	*Cyanobacteria*
JY101 & JY121	*Deinococcus-Thermus*
JY110 & JY121	*Chloroflexi*, *Planctomycetes*, *Spirochaetes*

Summary of common and unique bacterial phyla distributed among the four *Candida* spp. The comparison was assessed for yeast intra-genera analysis using Venn diagram constructed with InteractiVenn web-based tool [28]. Data on bacterial phyla distributed in the yeast strains was obtained using mothur V. 1.41.1 [24, 25]. The table is an extension to Fig 3 Ib. JY101, *C .tropicalis*; JY103, *C. metapsilosis*; JY110, *C. intermedia*; JY121, *C. suratensis.*

Table S2. Common and unique bacterial taxa at genus level between *Candida spp.*

**Yeast Strain(s)**	**Bacterial Genera**
JY103	*Fusobacterium*
JY110	*Enterobacteriaceae* unclassified, *Bacilli* unclassified, *Proteobacteria* unclassified, *Gammaproteobacteria* unclassified, *Actinomyces*
JY121	*Planococcus*, *Salinicoccus*, *Planomicrobium*, *Exiguobacterium*, *Microbacterium*, *Brachybacterium*, *Caulobacter*, *Kocuria*, *Bacillus*, *Brevundimonas*, *Anaerobacillus*, *Planococcaceae* unclassified, *Delftia*, *Jeotgalicoccus*, *Rhizobiales* unclassified, Dietzia, *Micrococcaceae* unclassified, *Sphingomonadaceae* unclassified, *Microbacteriaceae* unclassified, *Alphaproteobacteria* unclassified, *Methylobacterium*, *Sporosarcina*, *Ruminococcaceae* unclassified, Caulobacteraceae unclassified, *Bacillaceae* 1 unclassified, *Rhodococcus*, *Bacteroidetes* unclassified, *Ralstonia*, *Comamonadaceae* unclassified
JY101	*Gemella*, *Pseudomonadaceae* unclassified
JY101, JY103, JY110 & JY121	*Actinomycetales* unclassified, Bacteria unclassified, *Corynebacterium*, *Propionibacterium*, *Staphylococcus*, *Streptococcus*
JY103 & JY110	*Firmicutes* unclassified, *Granulicatella*, *Lactobacillales* unclassified, *Veillonella*
JY103 & JY121	*Bacillales* unclassified, *Brevibacterium*
JY101, JY103 & JY121	*Acinetobacter*
JY101 & JY121	*Rhodobacteraceae* unclassified
JY110 & JY121	*Stenotrophomonas*

Details as in Table S1.

Table S3. Common and unique bacterial taxa at phylum level between *Candida tropicalis*

Yeast Strain (s)	Bacterial Phyla
JY101	*Deinococcus-Thermus*
JY107	TM7
JY125	*Spirochaetes*, *Synergistetes*
JY114	*Gemmatimonadetes*
JY114 & JY125	*Chloroflexi*
JY107, JY114 & JY125	*Acidobacteria*
JY113 & JY125	*Planctomycetes*
JY101, JY107, JY113 & JY114	*Fusobacteria*
JY107, JY113 & JY114	*Armatimonadetes*
JY101, JY107, JY108, JY113, JY114 & JY125	*Actinobacteria*, Bacteria unclassified, *Bacteroidetes*, *Firmicutes*, *Proteobacteria*

Summary of common and unique bacterial phyla distributed among six *Candida tropicalis* strains i.e., strains JY101, JY107, JY108, JY113, JY114 and JY125. Other details as in Table S1. The table is an extension to Fig S3 Ib.

Table S4. Common and unique bacterial taxa at genus level between *Candida tropicalis*

Yeast Strain(s)	Bacterial Genera
JY101	*Gemella*, *Pseudomonadaceae* unclassified
JY107	*Firmicutes* unclassified, *Veillonella*, *Finegoldia*
JY108	*Alcaligenes*, *Serratia*, *Xanthomonadaceae* unclassified, *Proteobacteria* unclassified, *Gammaproteobacteria* unclassified, *Achromobacter*
JY125	*Comamonadaceae* unclassified, *Dietzia*, *Methylobacterium*, *Planococcaceae* unclassified, *Janibacter*, *Micrococcus*, *Microbacteriaceae* unclassified, *Rhodococcus*, *Bacteroidetes* unclassified, *Caulobacteraceae* unclassified, *Micrococcaceae* unclassified, *Bacillaceae* 1 unclassified
JY113	*Actinomyces*, *Facklamia*
JY114	*Caulobacter*
JY108 & JY114	*Delftia*
JY114 & JY125	*Planococcus*, *Salinicoccus*, *Planomicrobium*, *Exiguobacterium*, *Kocuria*, *Brachybacterium*, *Variovorax*, *Microbacterium*, Sphingomonadaceae unclassified, *Brevundimonas*, *Anaerobacillus*, *Bacillus*, *Rhizobiales* unclassified
JY107 & JY125	*Brevibacterium*
JY107 & JY113	*Lactobacillales* unclassified, *Lactobacillus*
JY107 & JY108	*Granulicatella*
JY101 & JY108	*Rhodobacteraceae* unclassified
JY108 & JY113	*Enterobacteriaceae* unclassified, *Pseudochrobactrum*
JY101, JY107, JY108, JY114 & JY125	*Propionibacterium*
JY101, JY107 JY113 & JY125	*Corynebacterium*
JY101, JY107, JY108, JY113 & JY125	*Streptococcus*
JY101, JY107, JY108, JY113, JY114 & JY125	*Actinomycetales* unclassified, Bacteria unclassified, *Staphylococcus*
JY101, JY108, JY113, JY114 & JY125	*Acinetobacter*
JY108, JY113 & JY125	*Stenotrophomonas*
JY108, JY114 & JY125	*Bacillales* unclassified

Summary of common and unique bacterial genera distributed among six *Candida tropicalis* strains i.e., strains JY101, JY107, JY108, JY113, JY114 and JY125. Other details as in Table S1. The table is an extension to Fig S3 IIb.

Table S5. Common and unique bacterial taxa at phylum level between *Pichia spp.*

Yeast Strain(s)	Bacterial Phyla
JY129	*Chlorobi*, *Deinococcus-Thermus*
JY116 & JY129	SR1
JY129 & JY131	*Armatimonadetes*, TM7
JY105, JY112 & JY131	*Spirochaetes*
JY105, JY112, JY129 & JY131	*Verrucomicrobia*
JY105, JY112, JY116, JY129, JY131 & JY136	*Acidobacteria*, *Actinobacteria*, Bacteria unclassified, *Bacteroidetes*, *Firmicutes*, *Planctomycetes*, *Proteobacteria*
JY112, JY116, JY129, JY131 & JY136	*Cyanobacteria*
JY105, JY129, JY131 & JY136	*Fusobacteria*
JY112, JY116, JY131 & JY136	*Chloroflexi*
JY112, JY116 & JY131	*Gemmatimonadetes*

Summary of common and unique bacterial phyla distributed among six *Pichia sp* strains viz., *P. kudriavzevii* strains JY105, JY112, JY116, JY129 and JY131 and *P. kluyveri* JY136. Other details as in Table S1. The table is an extension to Fig 4 Ib. JY105, *P. kudriavzevii*; JY112, *P. kudriavzevii*; JY116, *P. kudriavzevii*; JY129, *P. kudriavzevii*; JY131, *P. kudriavzevii*; JY136, *P. kluyveri*.

Table S6. Common and unique bacterial taxa at genus level between *Pichia spp.*

Yeast Strain(s)	Bacterial Genera
JY105	*Actinomyces*, *Gammaproteobacteria* unclassified, *Granulicatella*, *Lactobacillales* unclassified, *Pseudomonadaceae* unclassified, *Xanthomonadaceae* unclassified
JY116	*Neisseriaceae* unclassified, *Bacillaceae* 1 unclassified, *Rothia*
JY136	*Weissella*, *Alcaligenes*
JY129	*Micromonosporaceae* unclassified
JY131	*Lachnospiraceae* unclassified, *Ralstonia*, *Ruminococcaceae* unclassified, *Bacteroidales* unclassified, *Dermabacteraceae* unclassified, *Oligella*, *Clostridiales* unclassified, *Porphyromonadaceae* unclassified, *Siphonobacter*
JY116 & JY131	*Sphingomonas*
JY116 & JY136	*Paracoccus*
JY116 & JY129	*Halomonas*
JY105 & JY131	*Firmicutes* unclassified, *Gemella*, *Proteobacteria* unclassified
JY112, JY116, JY129 & JY131	*Delftia*, *Bradyrhizobiaceae* unclassified, *Planococcaceae* unclassified, *Caulobacter*
JY116, JY129 & JY131	*Comamonas*
JY105, JY112, JY116, JY129, JY131 & JY136	*Actinomycetales* unclassified, *Alphaproteobacteria* unclassified, Bacteria unclassified, *Corynebacterium*, *Propionibacterium*, *Staphylococcus*, *Streptococcus*
JY112, JY116, JY129, JY131 & JY136	*Planococcus*, *Salinicoccus*, *Brachybacterium*, *Planomicrobium*, *Exiguobacterium*, *Microbacterium*, *Brevundimonas*, *Kocuria*, *Sphingomonadaceae* unclassified, *Anaerobacillus*, *Bacillus*, *Bacillales* unclassified, *Variovorax*, Rhizobiales unclassified, *Methylobacterium*, *Micrococcus*, *Stenotrophomonas*, *Microbacteriaceae* unclassified, *Acinetobacter*, Caulobacteraceae unclassified
JY105, JY112, JY129, JY131 & JY136	*Brevibacterium*
JY116, JY131 & JY136	*Rhodobacteraceae* unclassified, *Bacteroidetes* unclassified
JY112, JY116, JY131 & JY136	*Dietzia*, *Comamonadaceae* unclassified
JY112, JY116 & JY131	*Micrococcaceae* unclassified

Summary of common and unique bacterial genera distributed among six *Pichia* spp. viz., *P. kudriavzevii* strains JY105, JY112, JY116, JY129 and JY131 and *P. kluyveri* JY136. Other details as in Table S1. The table is an extension to Fig 4 IIb. JY105, *P. kudriavzevii*; JY112, *P. kudriavzevii*; JY116, *P. kudriavzevii*; JY129, *P. kudriavzevii*; JY131, *P. kudriavzevii*; JY136, *P. kluyveri*.
