# Supplementary material for: Uncovering the hidden bacterial ghost communities of yeast and experimental evidences demonstrates yeast as thriving hub for bacteria
Source: Sci Rep. 2021 Apr 30;11:9394. doi: 10.1038/s41598-021-88658-x (PMC8087679; doi:10.1038/s41598-021-88658-x)
Supplement: Supplementary file 5 — Supplementary Information 2. [file 41598_2021_88658_MOESM5_ESM.docx]

**Movie S1.** Time lapse confocal video of *C. tropicalis* representing bacterial like moving bodies (BLBs) in the yeast. Total magnification was 630X using Zeiss LSM 880 confocal microscope.

**Movie S2.** Time lapse confocal video of stained *C. tropicalis* representing bacterial like moving bodies (BLBs) in the yeast. Staining was performed using ViaGram kit where DAPI/SYTOX stained the yeast nucleus and mitochondria of yeast cell whereas Texas Red stained the BLBs. Total magnification was 630X using Zeiss LSM 880 confocal microscope.

**Movie S3.** Time series of localised mCherry tagged fluorescent *P. stutzeri* inside the *C. tropicalis*. Total magnification was 630X using Zeiss LSM 880 confocal microscope.
